# *P. falciparum* Modulates Erythroblast Cell Gene Expression in Signaling and Erythrocyte Production Pathways

**DOI:** 10.1371/journal.pone.0019307

**Published:** 2011-05-04

**Authors:** Pamela A. Tamez, Hui Liu, Amittha Wickrema, Kasturi Haldar

**Affiliations:** 1 Department of Biological Sciences, Center for Rare and Neglected Diseases, University of Notre Dame, South Bend, Indiana, United States of America; 2 Department of Medicine, University of Chicago, Chicago, Illinois, United States of America; Duke University, United States of America

## Abstract

Global, genomic responses of erythrocytes to infectious agents have been difficult to measure because these cells are e-nucleated. We have previously demonstrated that *in vitro* matured, nucleated erythroblast cells at the orthochromatic stage can be efficiently infected by the human malaria parasite *Plasmodium falciparum.* We now show that infection of orthochromatic cells induces change in 609 host genes. 592 of these transcripts are up-regulated and associated with metabolic and chaperone pathways unique to *P. falciparum* infection, as well as a wide range of signaling pathways that are also induced in related apicomplexan infections of mouse hepatocytes or human fibroblast cells. Our data additionally show that polychromatophilic cells, which precede the orthochromatic stage and are not infected when co-cultured with *P. falciparum,* up-regulate a small set of genes, at least two of which are associated with pathways of hematopoiesis and/or erythroid cell development. These data support the idea that *P. falciparum affects erythropoiesis at multiple stages during erythroblast differentiation*. Further *P. falciparum* may modulate gene expression in bystander erythroblasts and thus influence pathways of erythrocyte development. This study provides a benchmark of the host erythroblast cell response to infection by *P. falciparum*.

## Introduction

Nearly 250 million cases of malaria were reported in 2008 [1]. *Plasmodium* is the causative agent of malaria, and *P. falciparum*, one of four species that infects humans, is the most virulent. In addition to acute febrile illness, it can cause severe disease including cerebral malaria [2] and severe malarial anemia [3] that have high rates of fatality. Infection is established when sporozoite- stage parasites transmitted by the bite of an infected mosquito enter hepatocytes. The intracellular parasites develop into merozoites, which upon release from the liver, infect circulating, mature erythrocytes. The resulting blood stage infection is responsible for all of the symptoms and pathologies associated with malaria. Blood stage parasites are known to extensively remodel erythrocytes [4], [5] to create an environment conducive to nutrient acquisition, escaping the host immune system, and for replication. Although as many as ∼400 parasite effector molecules have been predicted [6], [7], most host cellular targets remain unknown. One major reason is that mature erythrocytes are e-nucleated and thus transcriptional changes cannot be utilized as an important first step in identifying the host cell’s adaptive response to infection by blood stage *P. falciparum*. Understanding this may additionally be important to delineating the extent to which the cellular response to blood stage malaria infection is shared by infections by other pathogens.

Although the mature erythrocyte is undoubtedly the major host cell infected by *P. falciparum*, prior results have suggested that nucleated erythroblasts can also be infected [8], [9], [10], [11]. We recently reported that nucleated erythroblasts matured *in vitro* can be efficiently infected by *P. falciparum* [10]. However this is limited to the orthochromatic cells that are undergoing terminal differentiation and shutting down cellular processes. Here we now show that orthochromatic stages as well as earlier stage polychromatophilic cells, when co-cultured with *P. falciparum*, mount transcriptional responses that can be analyzed for their potential contribution to both infection and disease.

## Results and Discussion

### *P. falciparum* induces up-regulation of host cell genes at terminal stages of erythroid development

**Figure 1:**
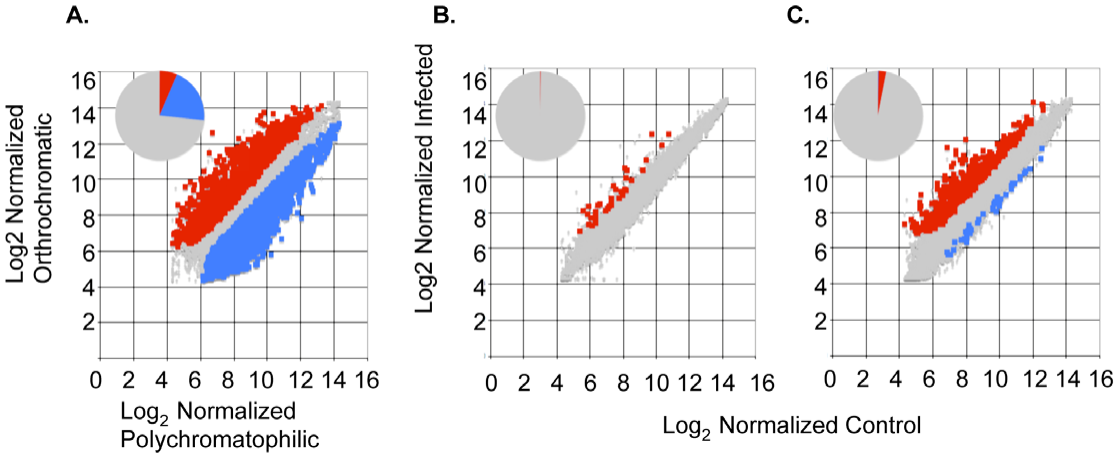
Transcriptional response of CD34+−derived hematopoietic stem cells to maturation and co-culture with *Plasmodium falciparum* 3D7 infection. A. Plot of log_2_ normalized signal from orthochromatic cells *vs* polychromatophilic cells. As cells differentiate, 5575 genes (26%) change (1342 up and 4233 down). B. Plot of log_2_ normalized signal from polychromatophilic cells co-cultured with *P. falciparum* 3D7 *vs* control, uninfected polychromatophilic cells. Only 35 genes (0.2%) are differentially expressed (all upregulated). C. Plot of log_2_ normalized signal from orthochromatic cells co-cultured with *P. falciparum* 3D7 *vs* control, uninfected orthochromatic cells. There are 609 genes modulated by infection; 570 are up- and 39 are down-regulated. Inset pie chart shows that malaria modulates ∼4% of the total transcripts. Up-regulated transcripts are denoted with red squares, down-regulated with blue squares, unchanged with grey diamonds.

In previous work we confirmed that the *in vitro* matured polychromatophilic and orthochromatic cells displayed expected stage specific progression of markers, indicating that they were undergoing proper erythroid differentiation [10]. Further, transcriptional analysis at a global level suggests that as many as 5575 genes (∼30%) display significant change, with the majority (4233 genes) being down-regulated as polychromatophilic cells differentiate into orthochromatic cells (Figure 1A).

We also demonstrated that 3D7 *Plasmodium falciparum* parasites efficiently infect orthrochromatic cells in culture. In contrast polychromatophilic cells are poorly infected, if at all [10]. Since both types of cells are in process of down-regulating cellular processes, we were interested in learning whether they mounted a measurable transcriptional response when co-cultured with *P. falciparum.* When polychromatophilic cells are co-cultured with *P. falciparum* (and less than 1% of cells are infected; Tamez, et. al. [10]) only 35 host genes are modulated, and all are up-regulated (Figure 1 B). In orthochromatic stages (where 60% of cells are infected; Tamez, et.al. [10]), 609 genes are differentially expressed, and 570 (94%) are up-regulated (Figure 1C). Thus in polychromatophilic cells *P. falciparum* exposure modulates 0.2% of expressed genes; while infection of orthochromatic cells modulates ∼4% of expressed genes. This is compared to 30% change in gene expression seen as the host cells differentiate from polychromatophilic to the orthochromatic stage. Together these data suggest that although transition from the polychromatophilic to the orthochromatic stage is accompanied by high levels of down-regulation of transcription, *P. falciparum* induces discrete, up-regulation of transcription in the host. Further infection *per se* does not directly alter the vast majority of global transcriptional responses at terminal stages of erythroid differentiation but may induce only a small subset of genes pertinent to interactions with the parasite.

### Co-culture and infection of orthochromatic stage cells with *P. falciparum* induces changes in a wide range of metabolic and signaling pathways

We began detailed analysis of co-cultures with orthochromatic cells, because these cells are infected and a substantial level of transcriptional changes is associated with infection. We utilized two different programs, Dchip [12] and GenePattern [13], to call differentially expressed genes in a comprehensive way, since the predictive rate of any given calling program can be limited and the relative overlap between genes called by each program is low. Next we used bioinformatic analyses to determine the biological processes that are enriched in these data. Since hundreds of host cell genes change when orthochromatic cells are co-cultured with *P. falciparum*, we limited ourselves to analyzing canonical pathways predicted by bioinformatics (rather than focusing on individual genes).

By Ingenuity Pathway Analysis (IPA), 14 canonical pathways represent the genes changed when orthochromatic cells are co-cultured with *P. falciparum* (Table 1, Supplementary Figure S1). IPA displays the statistical significance of each pathway, which is set to *p*<0.05 and is represented by the threshold −log(p-value) of 1.3. All pathways shown in blue bars are statistically significant and cross the threshold value of 1.3. Canonical pathways are based on the literature (journal article, KEGG pathways) and are used to overlay expression changes. One top pathway is the “NRF2-mediated Oxidative Response”, which delineates how cells neutralize reactive oxygen species. Another top pathway “Aryl Hydrocarbon Receptor Signaling” shows the cellular response when the receptor binds a polycyclic or halogenated hydrocarbon and subsequently activates transcriptional responses and downstream targets. c-Myc is a downstream target of the Aryl Hydrocarbon Receptor and promotes cell proliferation and tumorigenesis [14]. In addition to the top two canonical pathways, we also looked at “Biofunctions” data (Table 2, Supplementary Figure S2). Biofunctions are high-level biological categories populated by specific functions. All data is derived from the IPA Knowledge-Base, which associates array data with function rather than specific pathways. Both analyses suggest that upon infection, host cells are responding to accumulation of small molecules, such as reactive oxygen species and polyaromatic hydrocarbons. Numerous signaling pathways, additional metabolic and mitochondrial dysfunctional pathways are also manifest, suggesting that a wide range of canonical pathways may be involved in the response to infection.

**Table 1:** Genes associated with canonical pathways enriched in orthochromatic cells co-cultured with *P. falciparum*.

Ingenuity Canonical Pathways	Dchip molecules	GenePattern molecules
NRF2-mediated Oxidative Stress Response	GSR, ABCC1, STIP1, FOSL1, DNAJB1, ACTG1, CBR1	GSR, DNAJC21, HMOX1, JUN, STIP1, PIK3R1, DNAJA4, DNAJB1, ACTG1, CBR1
PPARalpha/RXRalpha Activation	*SMAD2*, HSP90AB1, ACOX1, PLCL2, NFKB2, GK	*SMAD2*, RELA, JUN, PRKAB1, HSP90AB1, TGFB1, ACOX1, GK, RXRA
Huntington's Disease Signaling	HSPA1A, HSPA6, DNAJB1, HSPA5, HSPA2	HSPA4, JUN, ARFIP2, HSPA1L, YKT6, HSPA1A, PIK3R1, HSPA6, CYCS (includes EG:54205), DNAJB1, CDK5R1
Glucocorticoid Receptor Signaling	*SMAD2*, IL8, HSP90AB1, HSPA1A, GTF2F2, HSPA6, FKBP4, NFATC1, PCK1, HSPA5, HSPA2	*SMAD2*, HSPA4, RELA, JUN, HSPA1L, HSP90AB1, HSPA1A, TGFB1, PIK3R1, HSPA6, FKBP4, PCK1
PXR/RXR Activation	*ABCB9*, ALAS1, CES2 (includes EG:8824)	RELA, ALAS1, CES2 (includes EG:8824), RXRA, ABCB9
Mitochondrial Dysfunction	GSR, TXN2, OGDH	*NDUFB2*, NDUFV2, GSR, GLRX2, TXN2, CYCS (includes EG:54205), OGDH
Lysine Biosynthesis	ALAS1, CCBL1	ALAS1, CCBL1
Cleavage and Polyadenylation of Pre-mRNA	CPSF6, CSTF3	CPSF6, CSTF3
Citrate Cycle	PCK1, OGDH	PCK1, IDH3A, OGDH
Rac Signaling		*ARPC5*, RELA, JUN, ARFIP2, PIK3R1, CDK5R1
Aryl Hydrocarbon Receptor Signaling	MYC, SRC, HSP90AB1, RARA, CDK6, NFKB2, ESR2, HSPB1	SRC, RELA, JUN, HSP90AB1, TGFB1, RARA, RXRA
TR/RXR Activation	KLF9, AKR1C1, AKR1C3, PCK1, THRB, AKR1C2	PIK3R1, PCK1, RXRA, AKR1C2
B Cell Activating Factor Signaling	NFATC1, IKBKAP, NFKB2	RELA, JUN
Polyamine Regulation in Colon Cancer	MYC, MXD1	

Genes in italics are down-regulated, all others are up-regulated.

**Table 2:** Genes associated with top biological processes when orthochromatic cells are co-cultured with *P. falciparum*.

Category	Dchip molecules	GenePattern molecules
Drug Metabolism	ADM, SLC7A11, AKR1C1, AKR1C3, UGCG, CES2 (includes EG:8824), GLRX, MYC, HSP90AB1, ABCC1, STIP1, FKBP4, DNAJB1, TFPI	SLC7A11, GLRX2, CES2 (includes EG:8824), GGT1, GSR, GAB1, HSPA1L, HSP90AB1, TGFB1, TXN2, STIP1, FKBP4, DNAJB1, RXRA, TFPI, ABCC4, GMFB
Endocrine System Development and Function	ADM, MYC, SRC, AKR1C1, HSP90AB1, AKR1C3, STIP1, FKBP4, DNAJB1, THRB	*IGF2*, ADM, SRC, HSP90AB1, HSPA1L, TGFB1, STIP1, FKBP4, DNAJB1
Lipid Metabolism	ADM, IL8, SRC, AKR1C1, AKR1C3, ACOX2, ACOX1, UGCG, PCK1, AKR1C2, GK, HSP90AB1, STIP1, ABCC1, FKBP4, DNAJB1, PRDX2	ADM, SRC, OXSM, ACOX1, PCK1, GK, LTB4R, HSPA1L, HSP90AB1, TGFB1, STIP1, FKBP4, CYCS (includes EG:54205), DNAJB1
Small Molecule Biochemistry	ADM, SRC, IL8, HTATIP2, AKR1C1, AKR1C3, ACOX2, ACOX1, UGCG, CES2 (includes EG:8824), PCK1, GK, AKR1C2, SYNJ2, HSP90AB1, STIP1, ABCC1, FKBP4, DNAJB1, TFPI, NANS, CCBL1, MCCC2, PRDX2	*IGF2*, ADM, SLC7A11, CLN8, CES2 (includes EG:8824), GK, HMOX1, LTB4R, HSPA1L, HSP90AB1, TGFB1, ALAS1, DNAJB1, GMFB, CCBL1, B4GALT5, SRC, OXSM, ACOX1, PCK1, GGT1, SYNJ2, PPOX, GAB1, STIP1, TXN2, FKBP4, CYCS (includes EG:54205), ALAS2, RXRA, TFPI, ABCC4
Cellular Compromise	IL8, MTSS1, HSPA1A, HSPH1, HSPA6, HSPA5, HSPA2, NBN, POLB, MYC, HSP90AB1, ABCC1, RARA, SERPINH1, HSPE1, DNAJB1, OGDH, HSPB1	*OPTN, TUBB*, GAS2, EHD1, SRC, YKT6, RRAD, HSPA1A, HSPH1, HSPA6, LMNA, CDK5R1, JUN, HSPA1L, HSP90AB1, TGFB1, GORASP2, RARA, SERPINH1, DNAJB1
Cellular Function and Maintenance	*SMAD2*, MRC1, SRC, HSPA1A, HSPH1, HSPA6, UGCG, HSPA5, HSPA2, JMJD6, HSP90AB1, RHOD, ABCC1, RARA, SERPINH1, MXD1, HSPE1, FKBP4, CFLAR, DNAJB1, ESR2, MCL1, HSPB1	*IGF2*, MRC1, SRC, HSPA1A, HSPH1, HSPA6, CCDC47, ACTG1, CDK5R1, JMJD6, JUN, HSPA1L, HSP90AB1, TGFB1, SERPINH1, RARA, NEB, FKBP4, DNAJB1, CFLAR, BCL2L11

Genes in italics are down-regulated, all others are up-regulated.

Alternative analysis of biological processes associated with gene changes seen in orthochromatic cells co-cultured with *P. falciparum* revealed top six functions shown in Table 3. Each process/category is broad and encompasses many discrete functions (each of which are used to generate p-values). Functions can be shared across processes /categories, and thus the function of “Binding of hormone/progesterone” is shared across four processes (“Drug Metabolism”, “Endocrine System Development and Function”, “Lipid Metabolism”, and “Small Molecule Biochemistry”; see Table 3). Similarly the function of “ER stress” is shared across two processes (“Cellular compromise”, “Cellular function and maintenance”). Notably several heat shock and DnaJ proteins are shared across the functions of hormone binding and ER stress, suggesting that a broad range of cellular changes are mediated by chaperones. The canonical pathways described in Table 1 are not amongst the top biofunctional categories, although heat shock and *dnaj* genes are prominent in both, again arguing that the host cell induces chaperones in response to infection. Our data provide the first evidence that erythroblasts at terminal stages of differentiation can respond transcriptionally to co-culture with *P. falciparum*. Our studies with orthochromatic cells greatly expand the list of host pathways that may potentially be involved in intracellular infection by *P. falciparum*.

**Table 3:** Genes associated with top biofunctions pathways in orthochromatic cells co-cultured with *P. falciparum.*

Functional Annotation	General Process	Dchip molecules	GenePattern molecules
binding of progesterone	Drug Metabolism, Endocrine System Dev. &Func., Lipid Metabolism, Small Molecule Biochemistry	DNAJB1, FKBP4, HSP90AB1, STIP1	DNAJB1, FKBP4, HSP90AB1, HSPA1L, STIP1
binding of hormone	Drug Metabolism, Endocrine System Dev. &Func., Lipid Metabolism, Small Molecule Biochemistry	DNAJB1, FKBP4, HSP90AB1, SRC, STIP1	DNAJB1, FKBP4, HSP90AB1, HSPA1L, SRC, STIP1
endoplasmic reticulum stress response of cells	Cellular Compromise, Cellular Function and Maintenance	DNAJB1, HSP90AB1, HSPA2, HSPA6, HSPA1A, HSPB1, HSPE1, HSPH1, RARA, SERPINH1	DNAJB1, HSP90AB1, HSPA6, HSPA1A, HSPA1L, HSPH1, RARA, SERPINH1
endoplasmic reticulum stress response	Cellular Compromise, Cellular Function and Maintenance	DNAJB1, HSP90AB1, HSPA2, HSPA5, HSPA6, HSPA1A, HSPB1, HSPE1, HSPH1, RARA, SERPINH1	CCDC47, DNAJB1, HSP90AB1, HSPA6, HSPA1A, HSPA1L, HSPH1, RARA, SERPINH1

Genes in italics are down-regulated, all others are up-regulated.

**Figure 2:**
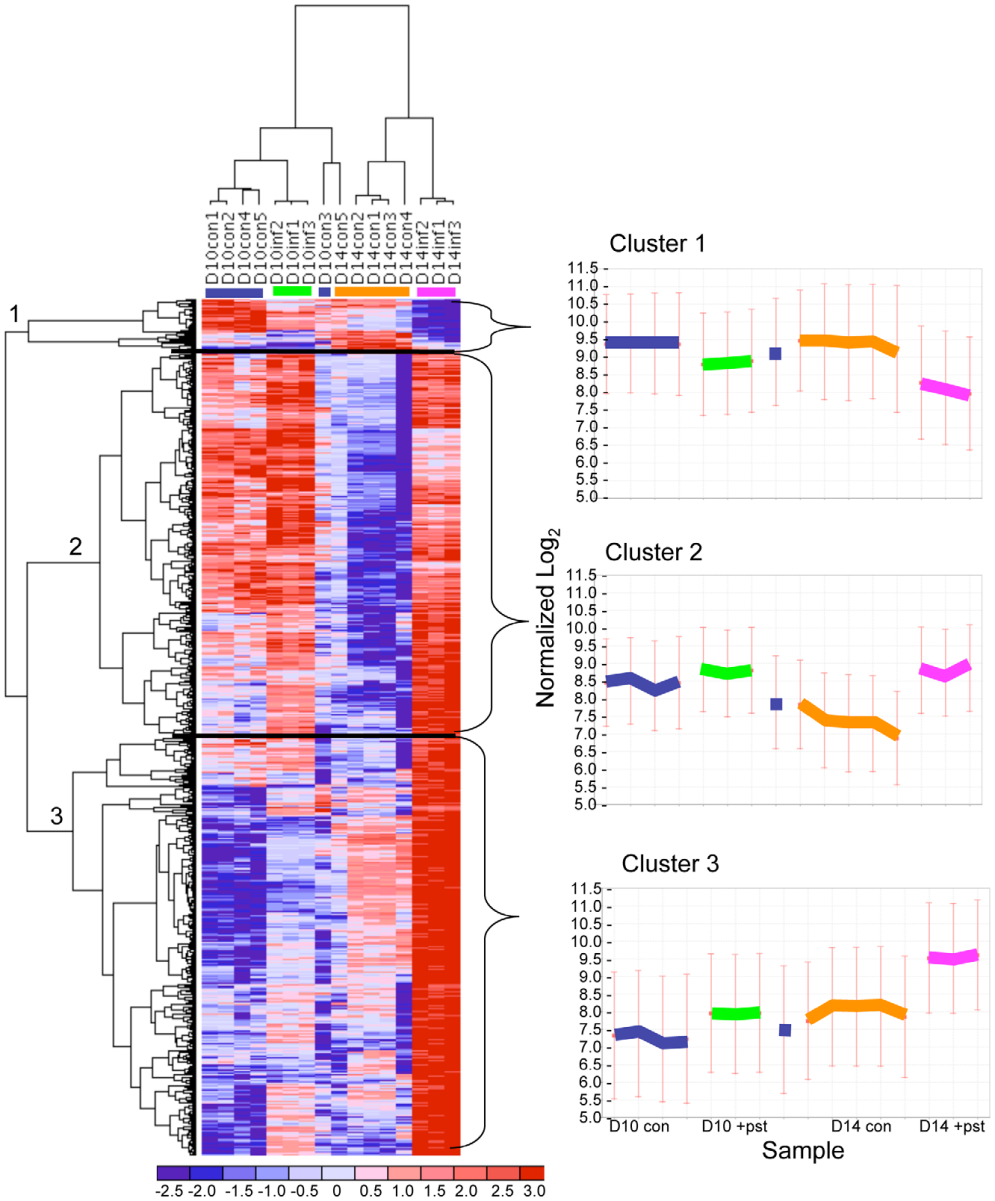
Heat map representing coordinated transcriptional changes. Genes are categorized into three main clusters, and a centroid plot showing normalized log_2_ values represents the expression of genes within each cluster. *The control Day 10 cells from donor 3 cluster more closely to D14 cells (lone blue bar), which may signify that they were more differentiated than the other Day 10 control samples. Fold change range is indicated from blue (negative) to red (positive).

**Figure 3:**
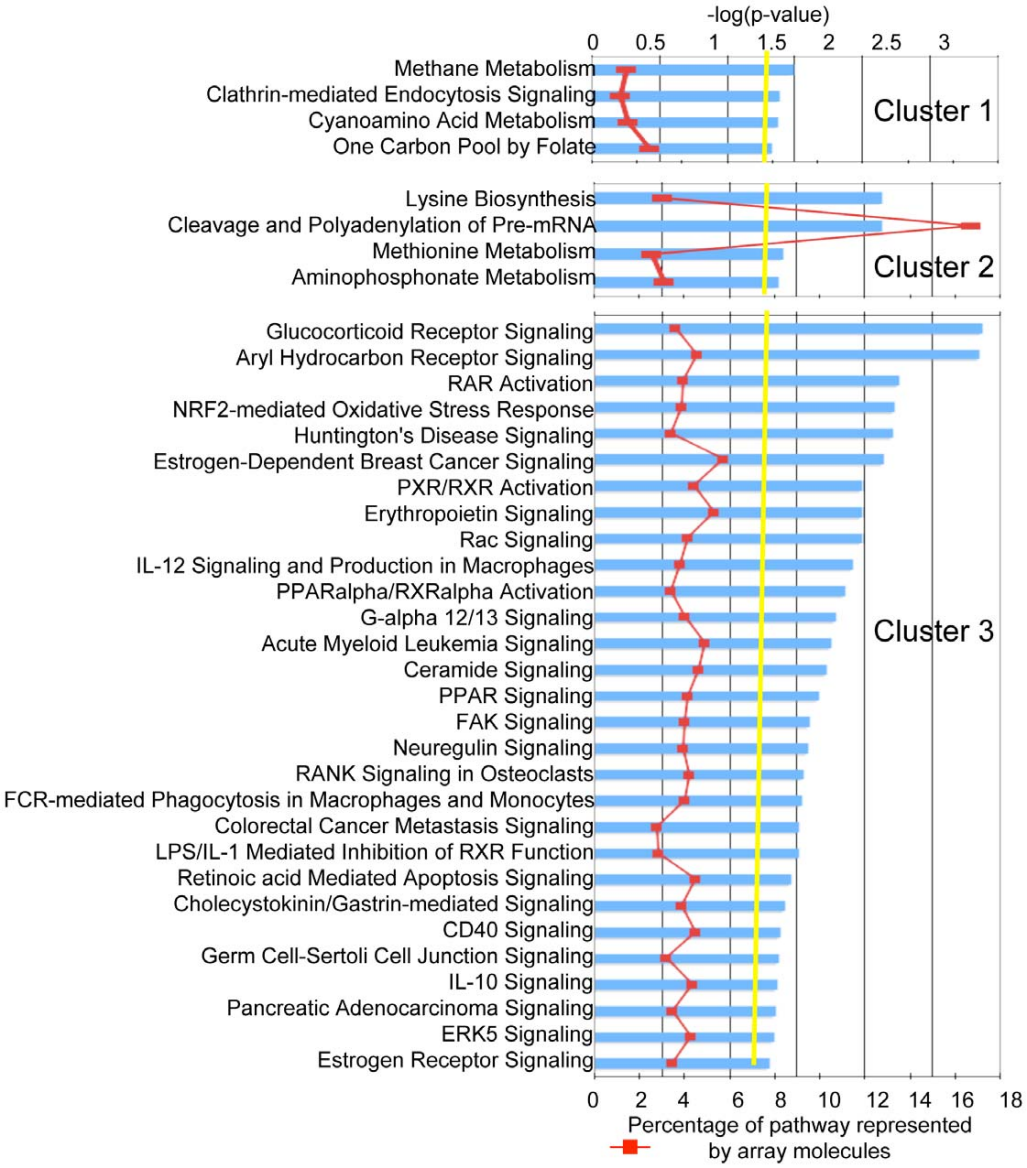
Pathways enriched in each of the three clusters. Because the input for cluster 1 was 39 genes, only three genes compromise all four enriched pathways (*shmt1*, *ap2b1*, *arpc5*). Cluster 2 is represented by metabolism and RNA modification; cluster 3 is represented by signaling pathways. Blue histograms illustrate statistical significance of p<0.5 (−log(p-value) >1.3), which is defined by the yellow “Threshold” line. The red line shows the number of array molecules as a percentage of the total molecules comprising that pathway.

To better understand the wide range of changes in gene expression in the context of the normal differentiation program, we clustered both the genes and arrays (Figure 2). Clustering of arrays confirmed that infected orthochromatic cells are more related to uninfected counterparts than to polychromatophilic cells and similarly, infected polychromatic cells are more closely related to uninfected counterparts than to orthochromatic cells. The genes that were changed (609 total) segregated into three main clusters: cluster 1 (39 genes) consists of genes down-regulated by malaria infection; cluster 2 (272 genes) consists of genes that are turning off as cells differentiate but turn on with malaria infection; and, cluster 3 (298 genes) consists of genes that are turning on as cells differentiate and were further induced by malaria infection (Figure 2). Thus, although most transcripts are up-regulated by malaria, there is no bias in affecting genes being turned on or off as cells mature. IPA analysis revealed that Cluster 1 is enriched in three metabolic pathways (all represented by *shmt1*) and “Clathrin-mediated Endocytosis Signaling” (represented by *ap2b1* and *arpc5*; see Figure 3). In addition to the statistical significance (indicated by the yellow line), IPA displays the proportion of each pathway that is represented by the array data (red line). In Cluster 1, since only three genes are associated and these appear to be a low proportion of genes in each pathway (<3%), we focused further detailed analysis on clusters 2 and 3.

**Figure 4:**
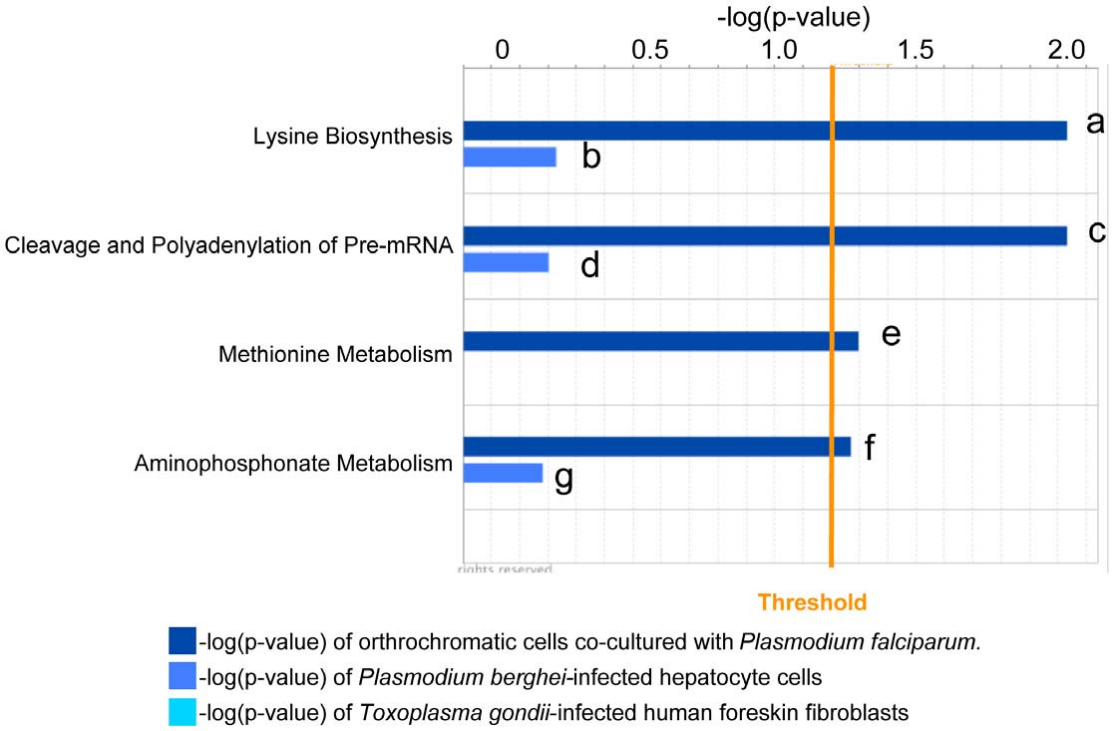
Comparative analysis of Cluster 2 data with infection with two organisms. All four pathways are specifically enriched upon co-culture of *P. falciparum* and orthochromatic cells. Dark blue histograms illustrate statistical significance of *Plasmodium falciparum-*infected orthrochromatic cells (p<0.5, −log(p-value) >1.3), which is defined by the orange “Threshold” line. Light blue histograms show significance levels of enriched pathways induced upon *Plasmodium berghei* infection of hepatocytes; cyan represents enrichment of pathways induced upon *Toxoplasma gondii* infection of human foreskin fibroblasts. Genes a, *alas1*,*ccbl1*; b, *vnn1*; c, *cpsf6*,*cstf3*; d, *cpsf6*; e, *alas1*,*ccbl1*; f, *alas1*,*ccbl1*; g, *pigf*, *mettl2b*.

By IPA analysis cluster 2 is enriched in four canonical pathways, two of which are metabolic and one “Cleavage and Polyadenylation of Pre-mRNA” (Figure 3). These genes are normally being down-regulated with differentiation, but infection with *P. falciparum* induces their gene expression. Pathways of lysine biosynthesis, methionine and aminophosphonate metabolism are associated with induction of two genes *alas1* and *ccbl1*. Cleavage and polyadenylation of Pre-mRNA is associated with *cpsf6* and *cstf3*, which appear to represent a high proportion of genes in that pathway (Figure 3 and 4). Neither set of genes is enriched in host cells (human foreskin fibroblasts) infected by a related apicomplexan *Toxoplasma gondii,* and neither is statistically significant in *Plasmodium berghei* (a rodent malaria parasite) infection of hepatocytes (Figure 4). One possibility is that these pathways may be specifically induced by *P. falciparum* co-culture with orthochromatic cells to provide nutrient or metabolic factors. The prediction that *P. falciparum* induces cleavage and polyadenylation of mRNA, lysine biosynthesis, methionine and aminophosphonate metabolism is completely unexpected. Since these pathways appear to be specific to *P. falciparum* infection, they need functional validation prior to further consideration.

**Figure 5:**
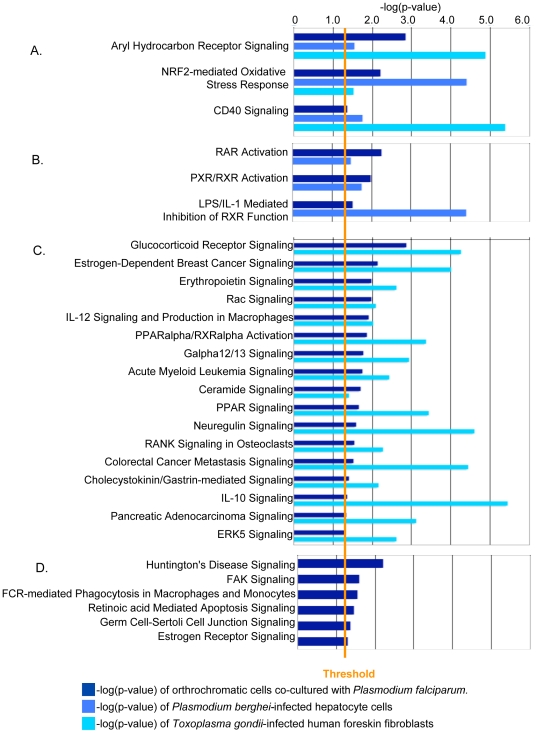
Comparative analysis of Cluster 3 data with infection by two other Apicomplexan organisms. Of the 29 pathways, (A) three are common to Apicomplexan infection; (B) three are unique to *Plasmodium* infection; (C) 17 describe Apicomplexan infection of human cells; and, (D) six are unique to *P. falciparum* infection of orthochromatic cells. Dark blue histograms illustrate statistical significance of *Plasmodium falciparum-*infected orthrochromatic cells (p<0.5, −log(p-value) >1.3), which is defined by the orange “Threshold” line. Light blue histograms show significance levels of enriched pathways induced upon *Plasmodium berghei* infection of hepatocytes; cyan represents enrichment of pathways induced upon *Toxoplasma gondii* infection of human foreskin fibroblasts.

Notably, cluster 3 is enriched in signaling pathways (Figure 3). Expression of these genes normally increases as cells differentiate, and they are further induced with *P. falciparum* co-culturing. The finding that 29 signaling pathways could be associated with infection of erythroblasts, was unexpected, since only the host Gs signaling pathway is currently associated with erythrocytic infection by *P. falciparum* [15]. Three of the pathways shown in Figure 3 “Aryl Hydrocarbon Receptor Signaling”, “NRF2-mediated Oxidative Stress Response”, and “CD40 Signaling” are common to infection of host cells by other apicomplexan parasites such as *Plasmodium berghei* infection of mouse hepatocytes as well as *T. gondii* infection of human foreskin fibroblasts (Figure 5A, Supplementary Table S1), suggesting that a wide range of host cells respond to apicomplexan infection by inducing pathways to detoxify polyaromatic hydrocarbons and reactive oxygen species as well as mount diverse cellular responses that can all be triggered by a TNF receptor family member (CD40).

Nuclear signaling through RAR and RXR is induced by just *P. berghei* and *P. falciparum* (Figure 5B, Supplementary Table S2). Additionally 17 of the 29 signaling pathways are shared by *T. gondii* and *P. falciparum* (Figure 5C, Supplementary Table S3). This suggests that the majority of the signaling events are not unique to *P. falciparum* infection of erythroid cells. This includes “Glucocorticoid Signaling” and “Erythropoietin Signaling” which are both important for erythropoiesis [15], [16]. However, since they are also seen in *T. gondii* infections, they are not expected to be uniquely correlated with malaria-specific disease processes. Of the six signaling pathways unique to *P. falciparum* infection, “Huntington’s Disease Signaling” is the lead pathway that is activated in response to protein aggregation and mediated by chaperones (Figure 5D and Table 4). These data are consistent with the concept that *P. falciparum* utilizes erythrocyte chaperones in erythrocytic infection. The induction of host chaperones lends strength to the idea that has emerged previously that erythrocyte heat shock proteins are needed for blood stage infection [17]. *P. falciparum* also expresses a wide range of heat shock and DnaJ proteins that are predicted to be exported to the host cell [18], suggesting that parasite and host chaperone functions may be important in host remodeling processes.

**Table 4:** Genes associated with top canonical pathways unique to orthochromatic cells co-cultured with *P. falciparum*.

Ingenuity Canonical Pathways	Molecules in Pfal infection of erythroid cells
Huntington's Disease Signaling	JUN, ARFIP2, HSPA1L, HSPA1A, PIK3R1, HSPA6, DNAJB1, CDK5R1
FAK Signaling	SRC, PIK3R1, TNS1, ACTG1
Fcgamma Receptor-mediated Phagocytosis in Macrophages and Monocytes	HMOX1, SRC, PIK3R1, ACTG1
Retinoic acid Mediated Apoptosis Signaling	RARA, CFLAR, RXRA
Germ Cell-Sertoli Cell Junction Signaling	SRC, TGFB1, PIK3R1, ACTG1, RAB8B
Estrogen Receptor Signaling	SRC, DDX5, H3F3B, PCK1

That additional *P. falciparum* –induced erythroid infection pathways share responses with other apicomplexan infections in mouse hepatocytes and human foreskin fibroblasts suggests that at least some of these transcriptional changes are broader responses linked to intracellular niche, such as nutrient uptake. Further Gs pathways involved in *P. falciparum* infection of erythrocytes are not upregulated in orthochromatic cells, and it should be noted that pathways can be targeted without eliciting a transcriptional response. Nonetheless, in the case of genes that are transcriptionally upregulated upon infection of orthochromatic cells, validation by knock downs of candidate pathways should reveal the extent to which blood stage malarial infection indeed mimics the paradigms of intracellular infection seen in a wide range of mammalian hosts.

Mounting an inflammatory response is an important antimicrobial response, but we identified only two genes induced upon orthochromatic co-culturing with *P. falciparum* that could potentially negatively regulate erythropoiesis, *jun* and *rara* (Supplementary Table S4). Jun has been reported to block erythroid differentiation by inhibiting GATA-1 activation through downstream effectors [19]. RARA activation can inhibit erythropoietin-induced erythroid colony formation [20], [21]. Eight molecules listed in Supplementary Table S4 play important roles for differentiating stem cells into other hematopoietic lineages. The significance of these molecules is unclear since 95% of the cell population used in this study is positive for Glycophorin A, an erythrocyte hallmark. In addition to these molecules, we looked for expression of cytokines, chemokines, and interleukins as a surrogate marker for the potential of these cells to mount an inflammatory response. Interestingly, most of the cytokines implicated in exacerbating malaria pathogenesis are not expressed in polychromatophilic or early orthochromatic cells: *ccl3* (*mip1a*), *ccl4* (*mip1b*), *ccl5* (*rantes*), *ifngamma, il12, il3, il4, il4r, il6, il1b, tnfalpha, tnfsf10* (*trail*). Only *clec11a* and *il1rn* (*il1ra*) are expressed but do not change upon co-culture with malaria. This suggests that later stage erythroid precursor cells have limited capacity to influence the extracellular environment, implicating a primary role for macrophages in determining the course of terminal differentiation. In future studies, it will be important to investigate the cellular source of cytokine production to determine whether macrophages are the primary source or whether early stage erythroblasts also contribute to production.

**Figure 6:**
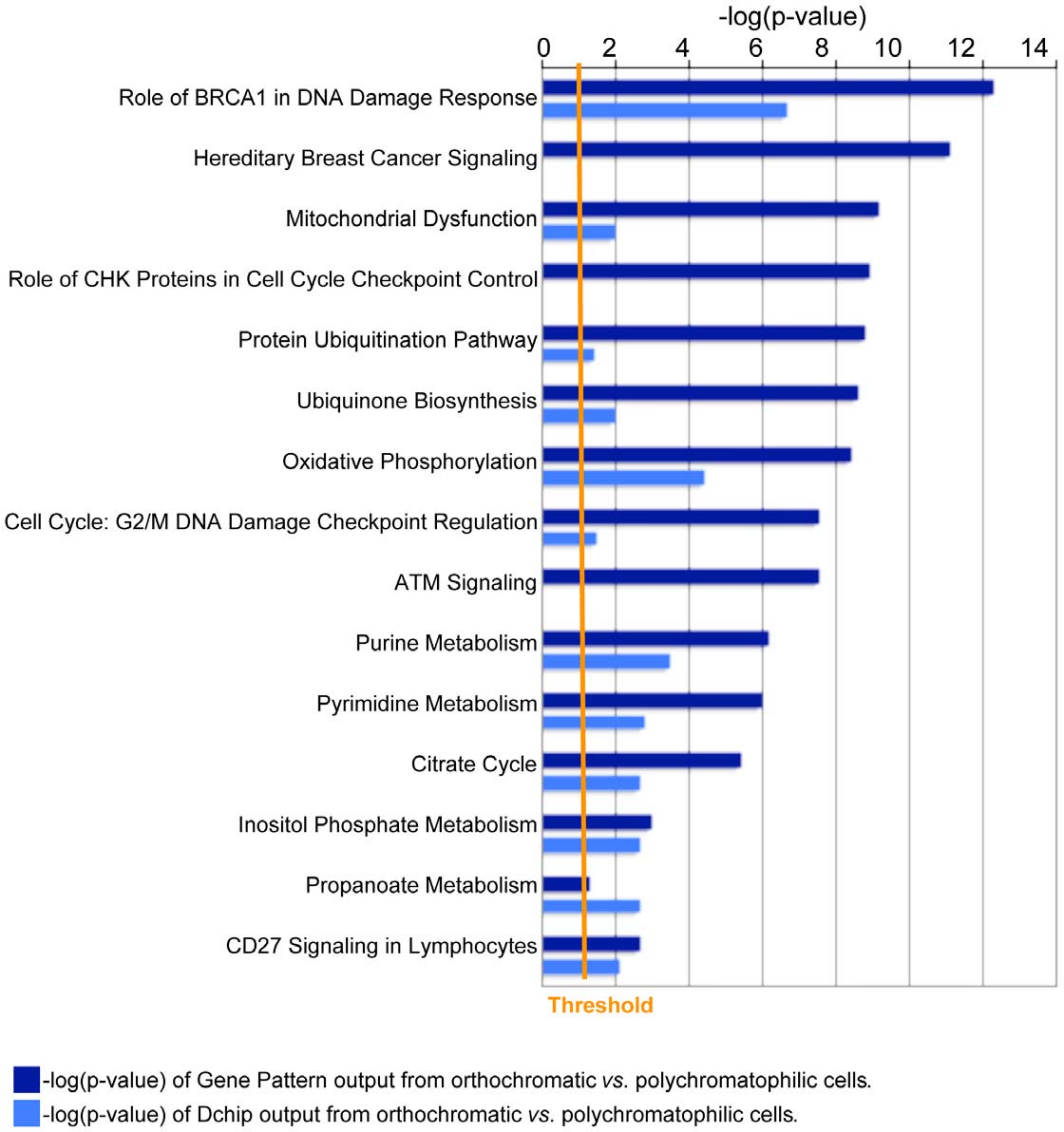
Pathways enriched as erythroblasts differentiate from polychromatophilic to orthochromatic cells. Only the top ten canonical pathways from GenePattern (dark blue bars) and Dchip (light blue bars) are displayed. For a full list, please see Supplementary Table S5. Histograms illustrate statistical significance of p<0.5 (−log(p-value) >1.3), which is defined by the orange “Threshold” line.

Since identifying the determinants of stage-specific susceptibility of erythroblasts to malaria infection is an important question, we also looked at the transcriptional profile of control orthochromatic cells as compared with control polychromatic cells. The associated pathways analyzed by IPA support that the major events are shutdown in signaling pathways associated with DNA repair and cell cycle, and in metabolic processes of oxidative phosphorylation and nucleotide metabolism (Figure 6, Supplementary Table S5). The data confirm that these cells are down regulating processes in anticipation of enucleation that precedes maturation to the reticulocyte stage. That the majority of processes are being down-regulated suggests that the parasite prefers an erythrocyte host with little to no metabolic and signaling activities. More validation will be required to hone in on specific molecular processes.

### Co-culture of polychromatophilic cells with *P. falciparum* induces changes in expression of host genes involved in developmental processes of red blood cells

In contrast to orthochromatic stages, polychromatic cells are not efficiently infected by *P. falciparum* but nonetheless up-regulate a maximum of 35 genes in co-cultures (Table 5). Because both the infection efficiency and transcriptional responses are low, it is possible that some of these changes are due to bystander effects or contamination with infected, later-stage cells. In order to rule out the second possibility, we restricted our analysis to the two genes that are called by both Dchip and GenePattern to be up-regulated in polychromatophilic co-cultures but that are not changed in orthochromatic co-cultures, *gdf15* and *tmem70.* Of these, only *gdf15* has been linked to malaria infection.

**Table 5:** List of genes changed in polychromatophilic cells co-cultured with *P. falciparum*.

Dchip output	GenePattern output
AHDC1, C11orf77, C14orf173, C17orf44, **GDF15**, hypothetical protein MGC32805, JUN, NBN, RRAD, **TMEM70**	BCL6, CDNA FLJ36202, CDNA FLJ90571, CFLAR, CLEC2B, DIDO1, EYA3, FOSL1, **GDF15**, H3F3A /// H3F3B, HCG27, HIST1H2BK, HIST2H2BE, hypothetical gene AK128882, Hypothetical LOC643837, hypothetical protein FLJ22639, hypothetical protein LOC284702, hypothetical protein LOC645676, INF2, NFYA, NKX3-1, NUP50, PIGL, RRAD, RXRA, SLC13A4, SOX6, SRXN1, **TMEM70**, TNFAIP3, TNS1, TOR1AIP1, Transcribed locus, Transcribed locus, ZCCHC2

All genes are up-regulated; text in bold shows common genes between the two analyses.

We argue that changes due to bystander effects are as important as direct effects and provide *gdf15* as an example. GDF15, a member of the TGFbeta family, is a cytokine that is secreted into the extracellular milieu and could potentially affect many neighboring cells in a paracrine manner. Notably, one study found elevated serum levels of GDF15 in malaria-infected and symptomatic patients [22]. However, GDF15 levels were not different between asymptomatic patients and healthy controls [22], suggesting that it may play a role in pathogenesis. Malaria infection is known to disrupt iron homeostasis [23], so investigating the interplay between GDF15 and hepcidin during malarial attacks could well be important.

GDF15 is thought to contribute to iron overloading, a correlate of ineffective erythropoiesis, by preventing hepcidin expression and thereby increasing iron absorption [24]. Thus it is possible that direct exposure of polychromatic cells to *P. falciparum* and its byproduct hemozoin (such as in the bone marrow), even in the absence of infection, may influence erythroid cellular development. In support of this, a recent study by Skorokhod and colleagues revealed that direct exposure of the earliest erythroid progenitor cells to hemozoin or 4-hydroxynonenal (one active component of hemozoin) for 1 day resulted in 30% reduction of cell growth [25]. Additionally, cells exit the cell cycle and arrest in G_0_/G_1_ phase. This is of interest since studies suggest that hemozoin and infected erythrocytes are present in the bone marrow where erythropoiesis occurs [26], [27], and dyserythropoiesis is thought to be associated with malarial anemia.

In conclusion, our data support the idea that *P. falciparum* and its byproducts may negatively affect erythropoiesis at multiple steps. Although early stage progenitors may not be susceptible to infection, they may be susceptible to the effects of by products such as hemozoin. We show that orthrochromatic cells induce a heat shock response when co-cultured with *P. falciparum*. It will be important to distinguish between direct and indirect effects on erythropoiesis as a consequence of parasite infection. We present data on erythroid cell transcriptional response during co-culture with malaria parasites *in vitro*. This response will be combined with effect of immune cells (because they secrete cytokines) during *in vivo* infection. Hence, our data provide a benchmark to separate effects of direct exposure to parasites from those of immune cells on erythroblasts.

## Materials and Methods

### Primary erythroid human cultures and parasite infection

Human primary erythroid cells, purchased from ALL Cells, Inc. (Emeryville, CA), were differentiated from CD34+ hematopoietic stem cells isolated from growth factor-mobilized peripheral blood as described [28]. Cells from five donors were cultured until polychromatophilic and orthochromatophilic stages of differentiation and served as uninfected control samples. Of the five donors, three were used to initiate *Plasmodium falciparum* (3D7) infection, which was performed as described [10]. Infected cells were harvested 24 hours post-infection, and RNA was isolated with Trizol (Invitrogen) and purified with RNeasy columns (QIAGEN) according to manufacturer recommendations. Microarray labeling and hybridizations were done according to Affymetrix protocols using HG U133 plus 2.0 chips.

### Transcriptional analysis

Statistical analysis was performed using the GenePattern suite of programs [13]. Data were normalized with RMA using the “ExpressionFileCreator” module and filtered using the default settings of “PreprocessDataset” module. Differential expression values, false discovery rates, and p-values were calculated with the “ComparativeMarkerSelection” module (test direction  = 2-sided, test statistic  =  T-test, number of permutations  = “0”, complete  =  no, balanced  =  no, smooth p values  =  yes). Differentially regulated transcripts with false discovery rates <0.05 were clustered using the “HierarchicalClustering” module after duplicates were removed. Both arrays and genes were clustered using pairwise complete linkage, and similarities calculated using Pearson correlation. Normalization and statistical analysis was also performed using Dchip [12] model-based expression, selecting genes with fold change >3 and p-values <0.05.

We used Ingenuity Pathway Analysis (Redwood City, California) to perform biological network analysis. Statistical significance was calculated using the Fisher’s exact test, and pathways with p-values <0.05 (−log(p-values) >1.3) were investigated. We analyzed both canonical pathways and bio-functions. Canonical pathways are validated biochemical and signaling pathways that have been published in textbooks and in the literature. These pathways are directional. Bio-functions are divided into three broad categories: Molecular and Cellular Functions; Physiological System Development and Function; and Diseases and Disorders. Lower-level functions, classified within these categories, are assigned p-values and come from findings within the Ingenuity Knowledge Base. They may be classified under many higher-level categories.

For comparative analyses with *Plasmodium berghei* infection of hepatocytes, we imported all genes listed in Additional File 1 from Lovegrove, et. al [29] into IPA. For *Toxoplasma gondii* infection of human foreskin fibroblasts, we imported genes listed in Figure 2 from Blader, et. al [30].

All data are available from GEO accession number GSE24849.

## Supporting Information

Figure S1 Canonical pathways enriched when orthochromatic cells are co-cultured with *P. falciparum* (associated with Table 1). Ingenuity Pathway Analysis shows 14 enriched canonical pathways, which represent the output from GenePattern (dark blue bars) and Dchip (light blue bars). Histograms illustrate statistical significance of p<0.5 (−log(p-value) >1.3), which is defined by the orange Threshold line.
(TIF)

Figure S2 Top six biological pathways, representing gene ontology and general physiological processes, are enriched upon *P. falciparum* co-culture with orthochromatic cells (associated with Table 2). GenePattern output is shown in dark blue bars; Dchip in light blue. Histograms illustrate statistical significance of p<0.5 (−log(p-value) >1.3), which is defined by the orange Threshold line.
(TIF)

Table S1 Genes associated with top canoncial pathways common to *P. falciparum, P. berghei* and *T. gondii* infection.
(XLS)

Table S2 Genes associated with top canoncial pathways common to *P. falciparum* and *P. berghei* infection.
(XLS)

Table S3 Genes associated with top canoncial pathways shared by *P. falciparum* and *T. gondii* infection of human cells.
(XLS)

Table S4 Genes associated with top immunological/inflammatory pathways when orthochromatic cells are co-cultured with *P. falciparum*.
(XLS)

Table S5 Genes associated with canonical pathways of orthochromatic erythroblasts *vs*. polychromatophiilic erythroblasts.
(XLS)
