# Supplementary figures and images for: P. falciparum Modulates Erythroblast Cell Gene Expression in Signaling and Erythrocyte Production Pathways
Source: PLoS One. 2011 May 4;6(5):e19307. doi: 10.1371/journal.pone.0019307 (PMC3087761; doi:10.1371/journal.pone.0019307)

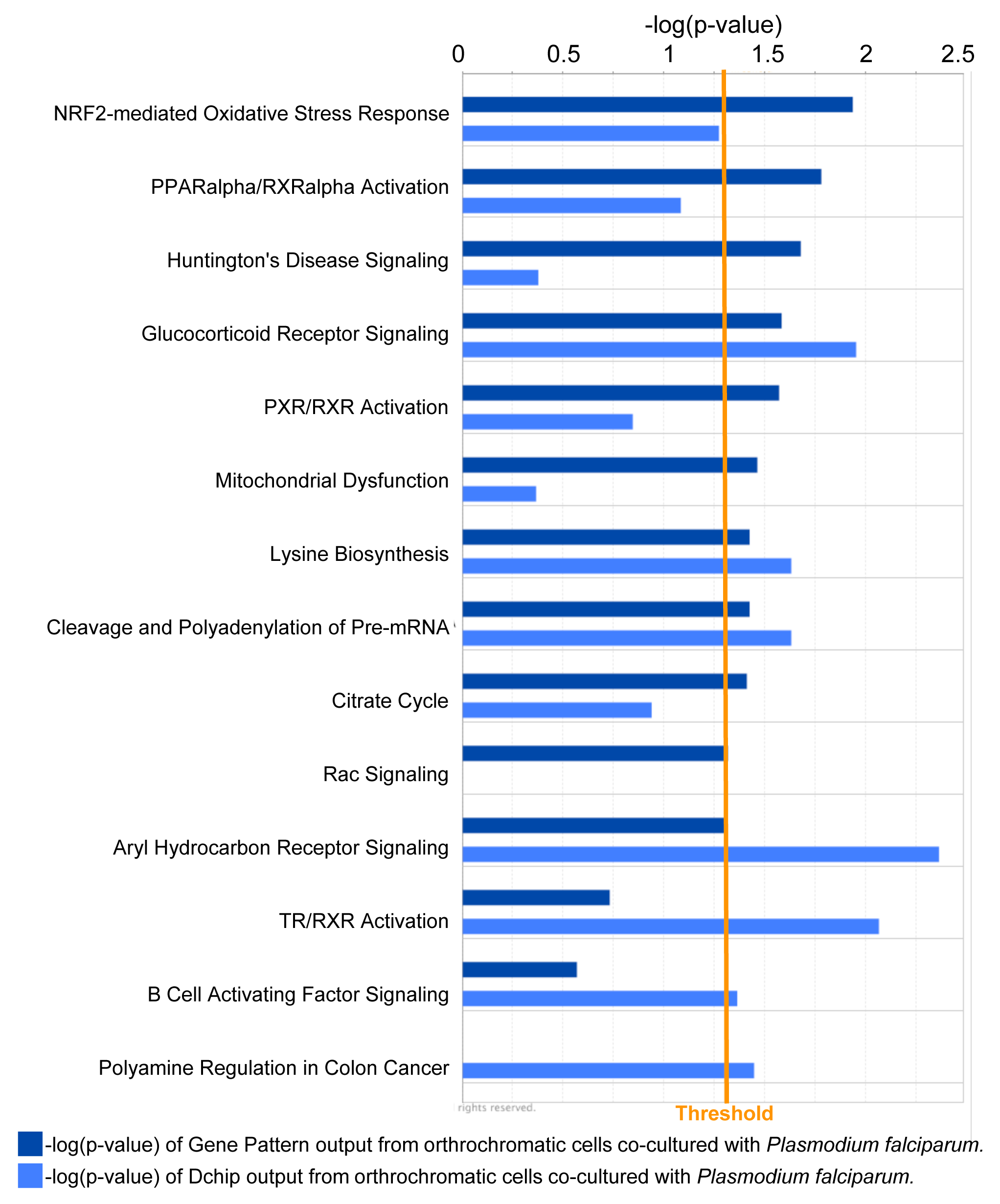

Supplement: Figure S1 — Canonical pathways enriched when orthochromatic cells are co-cultured with P. falciparum (associated with Table 1). Ingenuity Pathway Analysis shows 14 enriched canonical pathways, which represent the output from GenePattern (dark blue bars) and Dchip (light blue bars). Histograms illustrate statistical significance of p<0.5 (−log(p-value) >1.3), which is defined by the orange Threshold line. (TIF) [file pone.0019307.s001.tif]

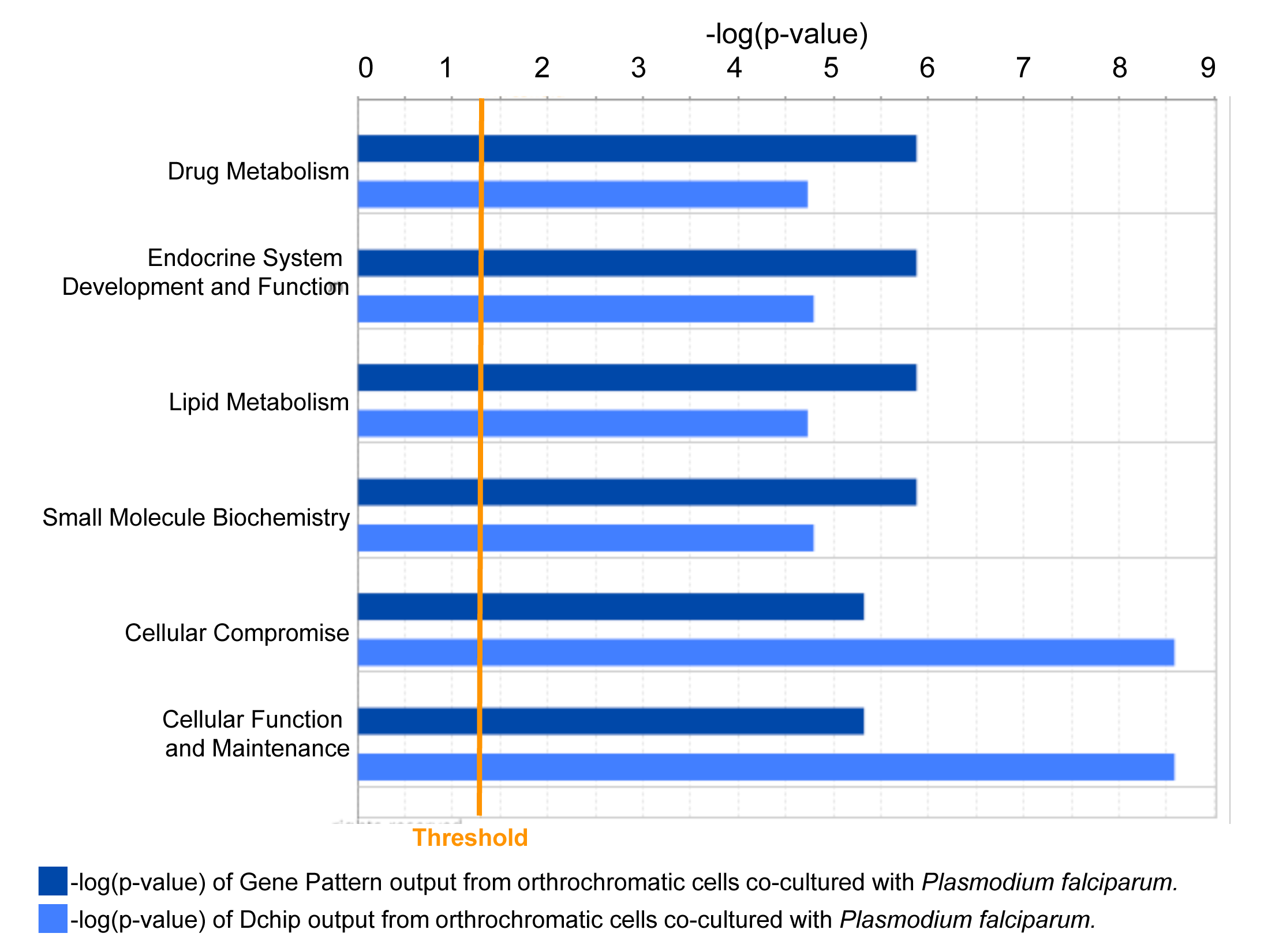

Supplement: Figure S2 — Top six biological pathways, representing gene ontology and general physiological processes, are enriched upon P. falciparum co-culture with orthochromatic cells (associated with Table 2). GenePattern output is shown in dark blue bars; Dchip in light blue. Histograms illustrate statistical significance of p<0.5 (−log(p-value) >1.3), which is defined by the orange Threshold line. (TIF) [file pone.0019307.s002.tif]
